# Evaluating the Link Between Thyroid Function Test Results and Levothyroxine Dose in the Management of Hypothyroidism: Can We Improve Dosing Regimes?

**DOI:** 10.1111/cen.70074

**Published:** 2025-12-22

**Authors:** Adrian H. Heald, Lakdasa D. Premawardhana, Peter N. Taylor, Suhani Bahl, Onyebuchi E. Okosieme, Anthony A. Fryer, Antonio Bianco, Colin M. Dayan, Michael P. Stedman

**Affiliations:** ^1^ The School of Medicine and Manchester Academic Health Sciences Centre University of Manchester Manchester England UK; ^2^ Department of Endocrinology and Diabetes Salford Royal Hospital Salford England UK; ^3^ Thyroid Research Group, Cardiff University School of Medicine Systems Immunity Research Institute Cardiff Wales UK; ^4^ School of Medicine Keele University Staffordshire England UK; ^5^ Medicine Endocrinology University of Chicago Chicago Illinois USA; ^6^ Res Consortium Andover England UK

**Keywords:** dose, free T4, hypothyroidism, Levothyroxine, treatment, TSH

## Abstract

**Introduction:**

Over 10 million thyroid function tests (TFTs) are carried out in England each year, most requests coming from primary care. Our previous work showed that only 25% of results for patients being treated with Levothyroxine fell within the TSH/FT4 boundary circumscribing 75% of untreated individuals. This study aimed to investigate further the differences in thyroid hormone levels, taking into account both diagnostic code and amounts prescribed.

**Methods:**

Using a city‐wide population record, we analysed TSH/FT4 simultaneous results from 47,869 consecutive diagnosed hypothyroid individuals by medication dose and 393,101 untreated/euthyroid individuals over 14 years. For those on medication, we only included those who were diagnosed over 2 years ago, had no more than two tests per year and more than 2 years of test results available. For those not on medication, we included results from those patients who had a single test or two tests with more than 4 years between tests.

**Results:**

The FT4 distribution for Levothyroxine‐treated individuals was similar in shape versus untreated individuals but shifted towards higher FT4 even at the lowest dose of Levothyroxine, with an increasing separation of the distributions as Levothyroxine dose increased (*F* value = 1.5 increasing to *F* value = 4.2). In contrast, the distribution of TSH was substantially different for untreated individuals versus those on Levothyroxine, where the distribution was massively skewed to low or undetectable TSH with a ‘hockey stick’ configuration, with increasing skewness as doses of Levothyroxine rose. For those not on thyroid hormone replacement, 90.3% of individuals were within the TSH reference range and of these, 0.8% were recorded with a low FT4. For those on medication, only 43.8% were within the TSH reference range. For men versus women, the median Levothyroxine dose was higher in all decades, with the highest median daily dose at age 50–59 years (men: 107 mcg/day; women 93 mcg/day). Median T4 rose (women > men) and TSH fell progressively (women > men) by age in treated individuals. The levels of TSH in treated and untreated populations were only similar at around FT4 = 20pmol/L: below this treated patients have a higher TSH and above it, treated have a lower TSH for the same FT4.

**Conclusion:**

We have here described that distribution of FT4/TSH is different in people on and off Levothyroxine treatment. For those on Levothyroxine, only 43.8% were within the TSH reference range and the degree of difference increased in treated individuals with Levothyroxine daily dose. The potential implication of our findings is that clinicians must be mindful as they diagnose and treat hypothyroidism that the administration of Levothyroxine, while in most but not all individuals is clinically beneficial does not return the individual to the same balance of TSH and FT4 as seen in euthyroid individuals.

## 1 Introduction

Hypothyroidism affects around 3% of the population in Europe [1] and nearly 5% of adult Americans (NIDDK) [2], being more common in females and older adults [3]. Clinically, hypothyroidism presents with symptoms such as not being able to bear the cold, tiredness and weight increase [4]. Biochemically, hypothyroidism is diagnosed through measurement of thyroid‐stimulating hormone (TSH) and free thyroxine (FT4), with increased TSH and decreased FT4 being the typical pattern seen in patients with overt hypothyroidism. Additionally, there exists a subset of the population with high TSH levels but normal FT4 and no or minimal symptoms; this is known as subclinical hypothyroidism [5]. The management of this has been discussed in a recent comprehensive review [6] and is addressed in NICE Guidance [7].

There are differences between the management of hypothyroidism in the United Kingdom versus the United States. In relation to major differences exist between the two health systems. For example, in the United States, many people with hypothyroidism will see an endocrinologist, whereas in the United Kingdom, hypothyroidism is largely managed by primary care physicians.

The most prescribed treatment for hypothyroidism is Levothyroxine monotherapy [6, 7], with this medication being converted into the more metabolically active thyroid hormone, triiodothyronine (FT3) [8], by deiodinases within the body. Levothyroxine is a very widely prescribed drug, with 33.8 million prescriptions in the United Kingdom alone in 2022 [9, 10]. Other thyroid hormone treatments, such as natural desiccated thyroid and Liothyronine, are available but are used much less commonly in modern practice [11].

The therapeutic goal in hypothyroidism is to restore clinical and biochemical euthyroidism via a physiologic thyroid hormone replacement. In most patients, Levothyroxine treatment will both normalise TSH levels and lead to symptom resolution. However, it is estimated that around 5%–15% of patients taking Levothyroxine still experience symptoms of hypothyroidism, even with normalised TSH levels [12, 13]. This may be because Levothyroxine monotherapy does not restore T3 levels to the normal range in a subset of individuals [14, 15], perhaps due to polymorphisms within the genes encoding peripheral deiodinase enzymes [11, 16].

Additionally, multiple studies [17, 18, 19] suggest that between 20% and 40% of patients taking Levothyroxine have TSH levels outside of the normal range, indicating potential under‐ or over‐replacement of thyroid hormones. Certain patient factors, such as sex, age and duration of treatment, may influence this under‐ or over‐treatment [20]. It is important to point out that both under‐ and over‐treatment of hypothyroidism are associated with increased all‐cause mortality [21, 22].

A better understanding of the relation between thyroid hormone replacement therapy and actual levels of TSH, FT3 and FT4 in patients could allow for a more tailored and effective therapy for people with hypothyroidism.

Over 10 million thyroid function tests (TFTs) are carried out in England each year, most requests coming from primary care with at least 59 million TFTs performed per year in the USA [23]. Our previous work showed that only 25% of results for patients being treated in one area of England fell within the TSH/FT4 boundary circumscribing 75% of untreated individuals [24].

This study aimed to investigate further the differences in thyroid hormone levels in people on thyroid hormone replacement therapy, taking into account, prescribed thyroid hormone replacement dose versus people being screened for thyroid disorder and not taking any Levothyroxine or other form of thyroid hormone replacement.

## 2 Materials and Methods

Retrospective analyses of primary care electronic health record (EHR) data from the Greater Manchester Care Record (GMCR) [25, 26] were undertaken. The GMCR pools EHR data for 2.85 million citizens across 433 general practices (99.67%) spread across the Greater Manchester conurbation. The base population is nearly everyone who resides in Greater Manchester. All primary care coded data including laboratory test results were available for analysis including SNOMED, CTV3 and ReadV2 codes [27, 28]. Patient data were pseudonymised at source and were extracted from the GMCR.

A) Laboratory results for TFT, patient ID, analysis date and test type (FT4, FT3 and TSH).
B) Prescribing data including medication including size, patient ID, prescription date and quantity.
C) Thyroid diagnosis data including the date and specific diagnosis.
D) Demographic data (ID, sex, date of birth, ethnicity and death) of those patients included above.

Data were coded using SNOMED, CTV3 and ReadV2 codes [27, 29]. The data were validated and cleaned prior to analysis by M. S.

### 2.1 Data Consolidation

The data set was consolidated:

a) Records were used for years 2010–2022 as assays were similar during this period.
b) Certain laboratories run a reflex testing strategy where TSH was first tested, and if outside reference range, the FT4 was then analysed. For this analysis, only those results where both TSH and FT4 were measured from the same sample were considered.
c) For those on medication, we only included those who had been diagnosed for more than 2 years and who had more than 2 years of test results available.
d) Patients whose diagnosis record was classified as ‘hypothyroid’ were included; patients whose diagnosis was classed as ‘thyroid disorder’ were excluded.

These test results were separated into two classes:

Untreated who had:

- No diagnosis of hypothyroid or thyroid disorder.
- No record of prescriptions for Levothyroxine.
- Only a single test or two tests with more than 4 years between tests (i.e., patients who were tested as part of a regular panel or diagnosis event).

Treated who had:

- First diagnosis for hypothyroidism more than 2 years ago, avoiding initial dose adjustments.
- Had prescriptions for Levothyroxine during that year.
- Had < 2 TFT tests in any calendar year (to exclude tests following dose adjustments).

For those on medication, the total prescribed amount in any calendar year was calculated by adding all prescription quantities together multiplied by tablet dose and dividing by 365 to give an average daily dose (ADD). These were then divided into four dose categories: lower 0–39/moderate 40–79/high 80–129/highest 130 or more mcg/day to reflect possible clinical need.

This study followed reporting instructions from RECORD (REporting of studies Conducted using Observational Routinely‐collected Data) guidelines [10].

Reference ranges were applied that had clinical significance, TSH reference range 0.4–4.0 miU/L and FT4 reference range 9–25pmol/L which covered all the local hospital FT4 reference ranges.

Data were consolidated into a set of tables with Excel Power Pivot and analysed, including the distribution of:

a) Numbers on T4 by Dose class mcg/day.
b) Numbers TSH and FT4 result values by those not on medication and on medication by daily dose class.
c) Variation of median values by sex and age class in treated and untreated.
d) The relationship between TSH and FT4 is non‐linear and generally regarded as an inverse logarithmic. To capture this effect we used the most commonly applied cutoff points (5%, 25%, 50%, 75% and 95%) to establish six ‘segments’ (< 5%, 5%–24%, 25%–49%, 50%–74%, 75%–94%, ≥ 95%) in both TSH and FT4 results and then examined how the other measure varies across these buckets by median value.

### 2.2 Ethics

The study was also reviewed and approved by the GMCR Expert Research Group [29] reference Number R 2023 065. The data used in the analyses presented were obtained with the permission of the GMCR Board and were fully anonymised prior to being made available to the investigators.

## 3 Results

Thyroid disease diagnosis codes were allocated into two classes, only those that had a diagnosis of hypothyroidism were included into this analysis (Table 1).

**Table 1:** Hypothyroid diagnoses.

INCLUDED Hypothyroidism	Acquired hypothyroidism, Subclinical hypothyroidism, Myxoedema, Postoperative hypothyroidism (excluding thyroid cancer), Irradiation hypothyroidism, Iodine hypothyroidism, Autoimmune hypothyroidism, Iatrogenic hypothyroidism, Post‐ablative hypothyroidism (excluding thyroid cancer), subclinical iodine deficiency hypothyroidism.
EXCLUDED Thyroid Disorder	Thyrotoxicosis, Hyperthyroidism, Graves disease, Thyroid nodule, Goitre, Disorder of thyroid gland, Simple goitre, Malignant tumour of thyroid gland, Non‐toxic multinodular goitre, Subclinical hyperthyroidism, Hashimoto thyroiditis, Thyroiditis, Cyst of thyroid, Non‐toxic nodular goitre, Toxic multinodular goitre, Carcinoma in situ of thyroid gland, Colloid goitre, Thyroid adenoma, Congenital hypothyroidism, Secondary hypothyroidism, Hypothyroidism in pregnancy.

A total of 87,673 individuals had a recorded diagnosis of hypothyroidism and 44,662 had a recorded diagnosis of thyroid disorder, that is, 3.8% of the total 2.3 million adult population of Greater Manchester was diagnosed with hypothyroidism at some point. In total, 485,074 results from 48,036 people were included into this analysis.

A total of 447,657 results from 391,577 untreated/euthyroid individuals who had been tested once or some twice with over 4 years between tests.

**Figure 1:**
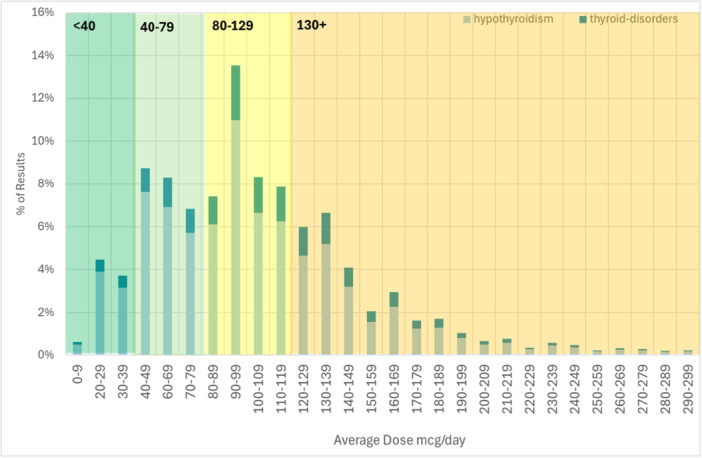
Distribution of Levothyroxine average daily dose (mcg) for 945,000 patient years split by diagnosis (note number of thyroid disorder diagnosis are not prescribed Levothyroxine).

The Levothyroxine levels were divided into four categories (Figure 1): 0–39 mcg, 19,834 patients with 77,526 results; 40–79 mcg, 190,136 results from 37,109 patients; 80–129 mcg, 131,066 results from 26,753 patients; and 130+ mcg, 13,076 results from 3149 patients. Overall, the most common dose range was 90–99 mcg/day and the median dose was 92 mcg/day.

Table 2 highlights how the TSH and FT4 values vary by group split by medication and diagnosis. It shows for the two groups of interest:

a) No Diagnosis/No Levothyroxine/Infrequent testing 391,577 patients; 447,657 results median TSH 1.7 (1.2–2.4) miU/L, FT4 median 14.4 (12.8–16.1) pmol/L ratio FT4/TSH 8.5.
b) Hypothyroid Diagnosis/Levothyroxine Medication/Stable testing 48,036 patients; 485,074 tests median TSH 2.46 (0.53–5.7) miU/L, FT4 15.6 (12.9–18.6) pmol/L ratio FT4/TSH 6.3.

**Table 2:** TSH and FT4 values and ratio for overall and various sub‐groups by diagnosis and Levothyroxine dose.

		TSH			FT4			
		Patients	Results	Median (IQR)	Patients	Results	Median (IQR)	FT4/TSH
Overall		1,135,751	7,647,173	1.76 (1.1–2.8)	1,116,183	5,154,852	14.4 (12.5–16.6)	8.2
Years 2010–2023		1,104,389	6,211,015	1.75 (1.1–2.77)	1,029,353	4,072,400	14.3 (12.5–16.4)	8.2
Data cleaned (in range and units)		1,102,397	6,120,743	1.74 (1.1–2.74)	1,025,875	3,998,812	14.3 (12.5–16.4)	8.2
Both TSH and FT4 avail		1,024,202	3,979,032	1.75 (1.1–2.8)	1,023,834	3,976,440	14.3 (12.5–16.4)	8.2
Medication	Prescribed Levothyroxine	90,713	810,132	2.4 (0.43–5.61)	90,912	823,983	15.3 (12.6–18.5)	6.4
	< 10 mcg/d or < 365 d	11,868	67,345	4.08 (1.88–6.3)	11,996	67,734	13.1 (11.2–15.4)	3.2
	No medication	921,621	3,101,555	1.7 (1.15–2.45)	920,926	3,084,723	14.2 (12.5–16)	8.4
No diagnosis	No medication	885,224	2,868,576	1.7 (1.2–2.42)	884,650	2,857,012	14.2 (12.5–16)	8.4
No diagnosis/no medication	Single or > 4 years	391,577	447,657	1.7 (1.2–2.4)	391,575	447,641	14.4 (12.8–16.1)	8.5
	Multiple tests	491,380	2,362,288	1.7 (1.2–2.42)	490,808	2,351,990	14.1 (12.5–16)	8.3
Diagnosis	Hypothyroidism	87,673	724,159	3.1 (0.87–6.1)	87,584	720,011	14.9 (12.3–18)	4.8
	Thyroid disorders	44,662	371,798	1.1 (0.16–2.91)	44,605	365,418	15.2 (12.9–18.4)	13.8
Hypothyroidism	No medication	9415	52,651	4.3 (2.5–5.9)	9396	52,361	13.6 (11.8–15.6)	3.2
	< 10 mcg/d or < 365 d	7756	42,131	5 (2.84–7.1)	7731	41,781	13 (11–15)	2.6
	Prescribed Levothyroxine	70,502	629,377	2.8 (0.67–6.03)	70,457	625,869	15.1 (12.5–18.2)	5.4
Hypothyroid with Levothyroxine	> 2 years and < 2 t/yr	48,036	485,074	2.46 (0.53–5.7)	48,033	482,528	15.6 (12.9–18.6)	6.3
	Initial or retesting	17,130	124,583	3.96 (1.23–6.9)	17,101	123,729	14 (11.6–17)	3.5
Levothyroxine dose	< 40 mcg/day	12,546	42,986	3.39 (1.91–5.7)	12,530	42,717	14 (11.7–16.4)	4.1
	40–79 mcg/day	27,946	121,524	2.65 (1.2–5.15)	27,915	120,884	15.2 (12.9–17.9)	5.7
	80–129 mcg/day	31,667	161,491	1.45 (0.27–4.51)	31,628	160,787	16.8 (14.1–19.6)	11.6
	130+ mcg/day	19,107	93,224	0.956 (0.15–4.98)	19,078	92,553	17.4 (14.6–20.5)	18.2

**Figure 2:**
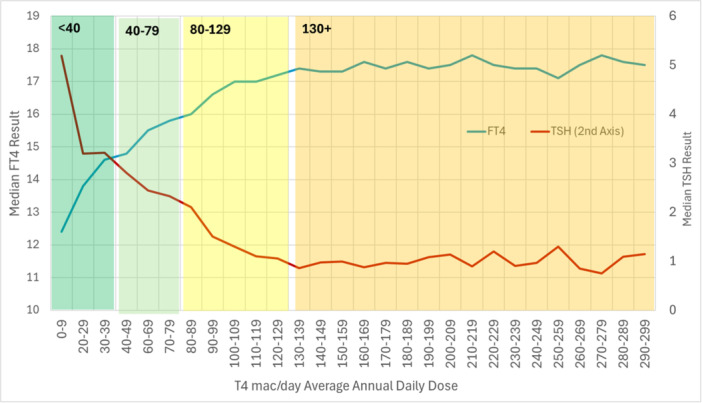
Population analysis linking average medication dose to median TSH and FT4 results.

Of the 447,657 results for untreated/euthyroid individuals, 90.3% (403,940) were within the TSH reference range (TSH‐RR) and 99.1% (443,461) in the FT4 reference range (FT4‐RR). Of the 406,311 results in the treated population, 177,957 (43.8%) were in the TSH reference range and 94.6% (384,301) were in the FT4 reference range. In treated hypothyroid individuals, the level of TSH fell and of FT4 rose markedly with increasing Levothyroxine dose (Figure 2).

**Figure 3:**
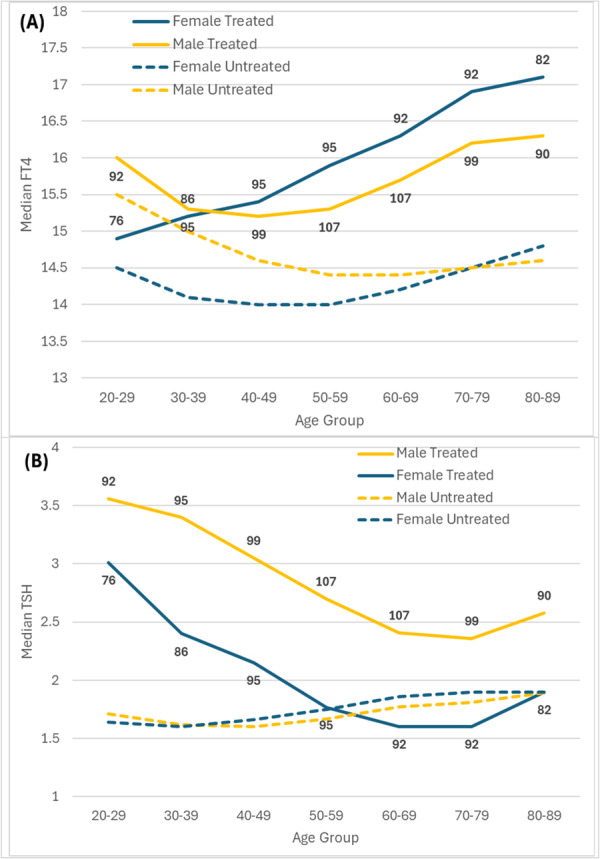
Variation difference in sex and age group between treated and untreated (A) FT4 and (B) TSH. *n *= average dose Levothyroxine mcg/day.

In treated individuals, for men versus women, the median Levothyroxine dose was higher in all decades, with the highest median daily dose at age 50–59 years (men: 107 mcg/day; women 93 mcg/day). Median FT4 rose with age of the patient (women > men) and TSH fell progressively (women > men) by age in treated individuals. In untreated individuals, there was much less variation by age decade for both men and women, but TSH did rise slightly with age in both sexes (Table 3 and Figure 3). In Table 3, the change in TSH and FT4 by age is seen as the age decade columns progress to the right.

**Table 3:** Splitting of dose and TFT result by age decade and sex including median Levothyroxine dose and median FT4 and TSH plus interquartile range (IQR) for (A) treated hypothyroid individuals, (B) untreated.

Age	20–29	30–39	40–49	50–59	60–69	70–79	80–89	Total
(A) Treated								
*Total patients*	2535	6590	12,063	17,840	20,146	16,836	8302	47,426
TFT results	10,700	30,662	54,711	83,717	96,046	81,666	37,627	395,129
Median Levothyroxine mcg/day	76	86	95	95	92	92	84	92
TSH median (IQR)	3.20 (1.10–6.67)	2.54 (0.66–5.87)	2.30 (0.46–5.50)	1.94 (0.30–5.02)	1.77 (0.31–4.36)	1.74 (0.34–4.02)	2.02 (0.49–4.40)	1.99 (0.36–4.83)
FT4 median (IQR)	15.0 (12.3–18.0)	15.2 (12.5–18.2)	15.3 (12.7–18.3)	15.8 (13.1–18.7)	16.2 (13.7–19.0)	16.7 (14.1–19.5)	17.0 (14.3–19.9)	16.0 (13.4–19.0)
*Female patients*	2033	5541	9936	14,562	16,285	13,553	6700	38,388
TFT results	8759	26,464	45,585	69,167	78,265	66,281	30,836	325,357
Median Levothyroxine mcg/day	74	84	93	93	92	92	82	92
TSH median (IQR)	3.10 (1.01–6.50)	2.40 (0.58–5.70)	2.19 (0.39–5.30)	1.80 (0.27–4.71)	1.61 (0.29–4.04)	1.60 (0.32–3.79)	1.90 (0.45–4.25)	1.84 (0.33–4.59)
FT4 median (IQR)	14.9 (12.2–17.9)	15.2 (12.5–18.1)	15.4 (12.7–18.3)	15.9 (13.2–18.8)	16.3 (13.8–19.2)	16.9 (14.3–19.7)	17.1 (14.5–20.0)	16.1 (13.5–19.0)
*Male patients*	502	1049	2127	3278	3861	3283	1602	9038
TFT results	1941	4198	9126	14,550	17,781	15,385	6791	69,772
Median Levothyroxine mcg/day	87	92	99	107	105	99	90	99
TSH median (IQR)	3.62 (1.40–7.23)	3.50 (1.27–6.90)	3.10 (0.93–6.41)	2.75 (0.68–5.90)	2.49 (0.56–5.40)	2.40 (0.60–4.92)	2.60 (0.78–5.06)	2.69 (0.72–5.60)
FT4 median (IQR)	16.0 (12.8–19.1)	15.2 (12.6–18.4)	15.1 (12.5–18.2)	15.2 (12.7–18.1)	15.7 (13.0–18.5)	16.2 (13.7–19.0)	16.2 (13.8–19.0)	15.7 (13.0–18.6)
(B) Untreated								
*Total patients*	77,203	79,169	75,535	62,735	42,495	24,483	9037	338,689
TFT results	79,841	82,419	79,224	66,041	44,483	25,640	9438	387,086
TSH median (IQR)	1.69 (1.20–2.40)	1.60 (1.14–2.30)	1.63 (1.15–2.30)	1.70 (1.20–2.43)	1.80 (1.22–2.63)	1.88 (1.25–2.80)	1.90 (1.20–3.00)	1.70 (1.19–2.40)
FT4 median (IQR)	14.9 (13.0–16.6)	14.6 (12.9–16.2)	14.3 (12.6–16.0)	14.2 (12.5–15.9)	14.3 (12.7–16.0)	14.5 (12.9–16.2)	14.7 (13.0–16.4)	14.5 (12.8–16.2)
*Female patients*	44,157	41,371	35,093	27,725	19,593	12,065	4931	168,241
TFT results	45,863	43,325	36,897	29,216	20,484	12,668	5147	193,600
TSH median (IQR)	1.65 (1.15–2.35)	1.60 (1.11–2.30)	1.67 (1.16–2.38)	1.75 (1.20–2.57)	1.86 (1.24–2.80)	1.90 (1.23–2.92)	1.90 (1.20–3.10)	1.70 (1.17–2.47)
FT4 median (IQR)	14.5 (12.9–16.0)	14.1 (12.6–15.8)	14.0 (12.4–15.5)	14.0 (12.4–15.5)	14.2 (12.7–15.9)	14.5 (12.9–16.1)	14.8 (13.1–16.5)	14.2 (12.6–15.8)
*Male patients*	33,046	37,798	40,442	35,010	22,902	12,418	4106	170,448
TFT results	33,978	39,094	42,327	36,825	23,999	12,972	4291	193,486
TSH median (IQR)	1.71 (1.21–2.41)	1.62 (1.17–2.30)	1.60 (1.14–2.25)	1.67 (1.19–2.34)	1.78 (1.21–2.50)	1.82 (1.27–2.66)	1.90 (1.25–2.87)	1.70 (1.20–2.39)
FT4 median (IQR)	15.5 (13.4–17.3)	15.0 (13.1–16.9)	14.6 (12.9–16.4)	14.4 (12.7–16.1)	14.4 (12.8–16.0)	14.5 (13.0–16.2)	14.6 (12.9–16.2)	14.8 (13.0–16.6)

### 3.1 Distribution of FT4 and TSH

**Figure 4:**
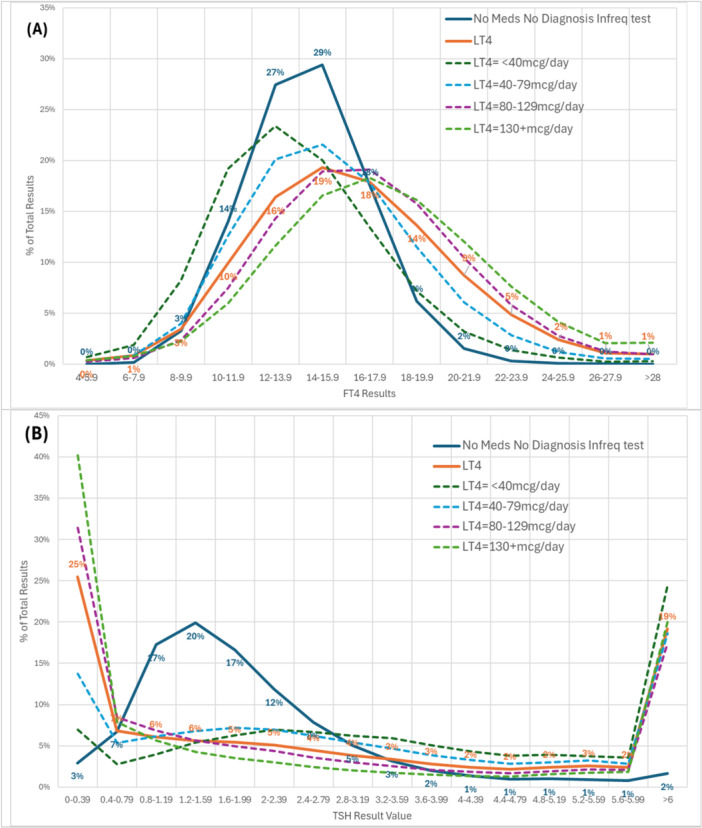
Distribution % of total results for untreated and treated (split by Levothyroxine dose/day) (A) FT4 and (B) TSH. The orange line represents all LT4‐treated patients.

The FT4 distribution for Levothyroxine‐treated individuals was similar in shape versus untreated individuals but shifted towards higher FT4 even at the lowest dose of Levothyroxine (Figure 4A), with an increasing separation of the distributions as Levothyroxine dose increased (*F* value = 1.5 increasing to *F* value = 4.2). There was a slightly higher degree of kurtosis for the untreated group. Of the 27% of individuals recording a low TSH, 10% of these were showing high FT4 values, suggesting overreplacement and potential for medication dose to be reduced, while 6% of those 31% recording high TSH had low FT4 values, with the potential to increase medication dose.

In contrast, the distribution of TSH was substantially different for untreated individuals versus those on Levothyroxine (Figure 4B). Specifically, the distribution of TSH for untreated individuals was broadly Gaussian, whereas the distribution of TSH for treated individuals was markedly skewed to the lower end of TSH, even for those on low daily doses of Levothyroxine with a ‘hockey stick’ configuration.

For those on medication, only 43.8% were within the TSH reference range. This effect was apparent even in lowest dose patients (52.3%) and was most apparent in highest dose patients (32.0%). As the Levothyroxine dose increased, the proportion of individuals below the TSH reference range increased (Figure 4B). In the non‐treated individuals, 90.3% of the TSH level were within the reference range.

Of the 25.5% of individuals recording a low TSH, 10.0% of these were showing high FT4 values suggesting overreplacement, with the potential for the medication dose to be reduced, while 65.6% of those 31.0% recording high TSH had low FT4 values, with the potential to increase medication dose.

**Figure 5:**
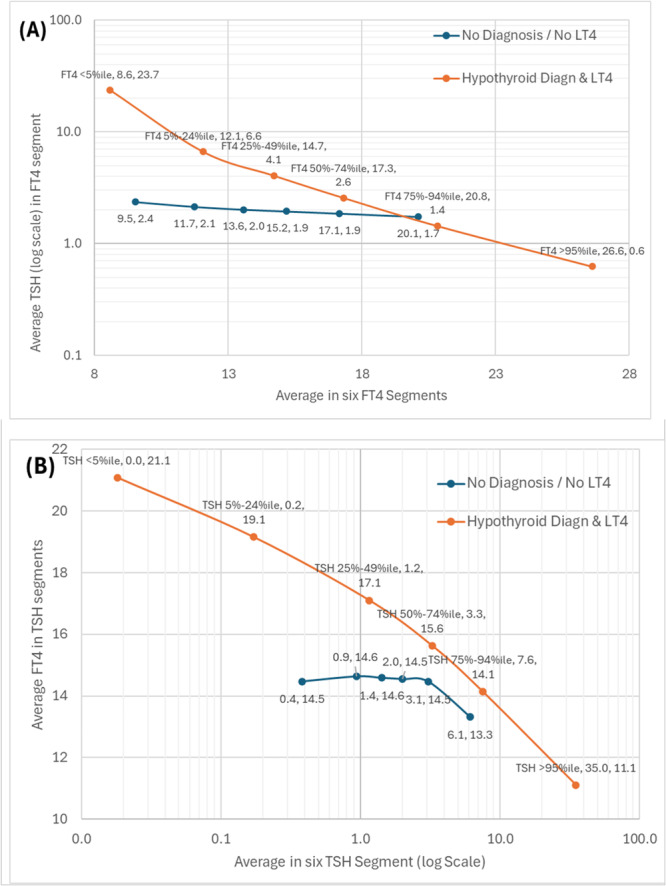
TSH linked to FT4 in treated and untreated population (A) FT4 segments segment with average TSH (B) TSH segments with average FT4. The relationship between TSH and FT4 is non‐linear and generally regarded as an inverse logarithmic. To capture this effect we used the most commonly applied cutoff points (5%, 25%, 50%, 75% and 95%) to establish six ‘segments’ denoted by each circle (< 5%, 5%–24%, 25%–49%, 50%–74%, 75%–94%, ≥ 95%) in both TSH and FT4 results and then examined how the other measure varies across these buckets by median value.

Figure 5A shows how the mean TSH values vary between the FT4 segments. It highlights that in the untreated population, the TSH is very stable, falling from 2.4 to 1.7 miU/L over the 90 centiles of FT4, increasing from 9.5 to 20.1 pmnol/L. While in the treated population, TSH falls from 23.7 to 0.6 miU/L as the FT4 increases from 8.6 to 26.6 pmol/L. This is in keeping with a substantially different balance of circulating FT4 versus TSH in people being treated with Levothyroxine versus people not taking Levothyroxine when TSH level is stratified by FT4.

The levels of TSH in treated and untreated populations were only similar at around FT4 = 20 pmol/L: below this, treated have higher TSH, and above it, treated have a lower TSH for the same FT4. The untreated population has a restricted range of TSH with a flat relation between FT4 and TSH. The treated population has a wide range of TSH with both 5%–25% and 75%–95% median values falling outside the < 5% and ≥ 95% untreated medians, and has a steeper inverse link between FT4 and TSH.

Figure 5B shows how the FT4 varies between the TSH segments with a similar difference between treated and untreated to TSH. This is a ‘mirror image’ of Figure 5B and demonstrates the same phenomenon but with stratification of FT4 by TSH.

**Figure 6:**
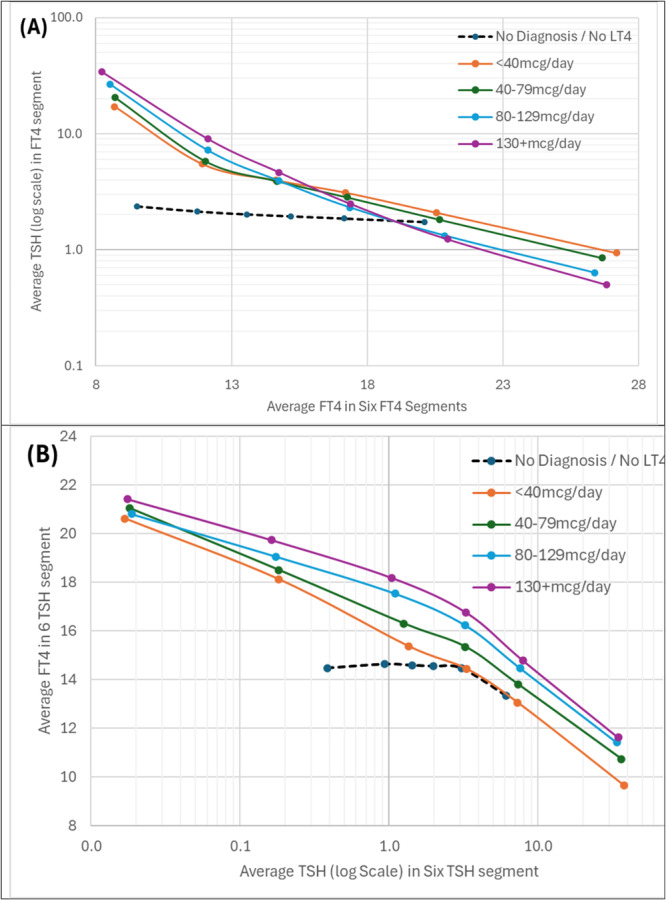
Segmented analysis with the treated values split by daily dose (A) FT4 segment (B) TSH segments. The relationship between TSH and FT4 is non‐linear and generally regarded as an inverse logarithmic. To capture this effect we used the most commonly applied cutoff points (5%, 25%, 50%, 75% and 95%) to establish six ‘segments’ denoted by each circle (< 5%, 5%–24%, 25%–49%, 50%–74%, 75%–94%, ≥ 95%) in both TSH and FT4 results and then examined how the other measure varies across these buckets by median value.

Figure 6A shows that even at the lower Levothyroxine doses the TSH values stratified by FT4 intervals are different from untreated individuals and that the slope for the curve increasing by Levothyroxine dose. A ‘mirror image’ of this pattern for FT4 stratified by TSH intervals is demonstrated in Figure 6B.

Tables 4 and 5 show how the combined FT4 and TSH values for each patient's results fell within the respective reference ranges. In Table 4, the contrast is between those on and off Levothyroxine treatment and in Table 5, we report the association with Levothyroxine daily dose.

**Table 4:** Link individual TSH and FT4 results each falling into respective reference range split by untreated and treated and by dose (% of total results/% TSH sub‐total/% FT4 sub‐total).

	High TSH: ≥ 4	Mid TSH: 0.4–3.9	Low TSH: < 0.4	Total
*Untreated (no diagnosis/no medication/few tests)*				
Total	30,603 (6.8%/100%/6.8%)	403,940 (90.3%/100%/90.3%)	12,864 (2.9%/100%/2.9%)	447,407 (100%/100%/100%)
Low FT4: < 9	395 (0.1%/1.3%/10.7%)	3263 (0.7%/0.8%/88.2%)	40 (0%/0.3%/1.1%)	3698 (0.8%/0.8%/100%)
Mid FT4: 9–24.9	30,203 (6.8%/98.7%/6.8%)	400,508 (89.5%/99.2%/90.3%)	12,750 (2.8%/99.1%/2.9%)	443,461 (99.1%/99.1%/100%)
High FT4: ≥ 5	5 (0%/0%/2%)	169 (0%/0%/68.1%)	74 (0%/0.6%/29.8%)	248 (0.1%/0.1%/100%)
*Treated (diagnosis hypothyroidism/prescribed levothyroxine/only annual test)*				
Total	124,858 (30.7%/100%/30.7%)	177,957 (43.8%/100%/43.8%)	103,496 (25.5%/100%/25.5%)	406,311 (100%/100%/100%)
Low FT4: < 9	7015 (1.7%/5.6%/82.2%)	1342 (0.3%/0.8%/15.7%)	180 (0%/0.2%/2.1%)	8537 (2.1%/2.1%/100%)
Mid FT4: 9–24.9	117,536 (28.9%/94.1%/30.6%)	173,737 (42.8%/97.6%/45.2%)	93,028 (22.9%/89.9%/24.2%)	384,301 (94.6%/94.6%/100%)
High FT4: ≥ 25	307 (0.1%/0.2%/2.3%)	2878 (0.7%/1.6%/21.4%)	10,288 (2.5%/9.9%/76.4%)	13,473 (3.3%/3.3%/100%)

**Table 5:** Link treated individual TSH and FT4 results each falling into respective reference range split by Levothyroxine average daily dose (% of total results/% TSH sub‐total/% FT4 sub‐total).

	High TSH: ≥ 4	Mid TSH: 0.4–3.9	Low TSH: < 0.4	Total
< 40 mcg/day	16,268 (40.5%/100%/40.5%)	20,978 (52.3%/100%/52.3%)	2896 (7.2%/100%/7.2%)	40,142 (100%/100%/100%)
Low FT4: < 9	1250 (3.1%/7.7%/71.3%)	479 (1.2%/2.3%/27.3%)	25 (0.1%/0.9%/1.4%)	1754 (4.4%/4.4%/100%)
Mid FT4: 9–24.9	14,999 (37.4%/92.2%/39.5%)	20,388 (50.8%/97.2%/53.6%)	2620 (6.5%/90.5%/6.9%)	38,007 (94.7%/94.7%/100%)
High FT4: ≥ 25	19 (0%/0.1%/5%)	111 (0.3%/0.5%/29.1%)	251 (0.6%/8.7%/65.9%)	381 (0.9%/0.9%/100%)
40–79 mcg/day	40,692 (33.8%/100%/33.8%)	63,178 (52.5%/100%/52.5%)	16,471 (13.7%/100%/13.7%)	120,341 (100%/100%/100%)
Low FT4: < 9	2081 (1.7%/5.1%/78.6%)	504 (0.4%/0.8%/19%)	63 (0.1%/0.4%/2.4%)	2648 (2.2%/2.2%/100%)
Mid FT4: 9–24.9	38,541 (32%/94.7%/33.3%)	62,124 (51.6%/98.3%/53.7%)	15,037 (12.5%/91.3%/13%)	115,702 (96.1%/96.1%/100%)
High FT4: ≥ 25	70 (0.1%/0.2%/3.5%)	550 (0.5%/0.9%/27.6%)	1371 (1.1%/8.3%/68.9%)	1991 (1.7%/1.7%/100%)
80–129 mcg/day	43,890 (27.4%/100%/27.4%)	66,399 (41.5%/100%/41.5%)	49,812 (31.1%/100%/31.1%)	160,101 (100%/100%/100%)
Low FT4: < 9	2195 (1.4%/5%/87%)	268 (0.2%/0.4%/10.6%)	61 (0%/0.1%/2.4%)	2524 (1.6%/1.6%/100%)
Mid FT4: 9–24.9	41,575 (26%/94.7%/27.4%)	64,822 (40.5%/97.6%/42.7%)	45,416 (28.4%/91.2%/29.9%)	151,813 (94.8%/94.8%/100%)
High FT4: ≥ 25	120 (0.1%/0.3%/2.1%)	1309 (0.8%/2%/22.7%)	4335 (2.7%/8.7%/75.2%)	5764 (3.6%/3.6%/100%)
130+ mcg/day	24,008 (28%/100%/28%)	27,402 (32%/100%/32%)	34,317 (40%/100%/40%)	85,727 (100%/100%/100%)
Low FT4: < 9	1489 (1.7%/6.2%/92.4%)	91 (0.1%/0.3%/5.6%)	31 (0%/0.1%/1.9%)	1611 (1.9%/1.9%/100%)
Mid FT4: 9–24.9	22,421 (26.2%/93.4%/28.5%)	26,403 (30.8%/96.4%/33.5%)	29,955 (34.9%/87.3%/38%)	78,779 (91.9%/91.9%/100%)
High FT4: ≥ 25	98 (0.1%/0.4%/1.8%)	908 (1.1%/3.3%/17%)	4331 (5.1%/12.6%/81.2%)	5337 (6.2%/6.2%/100%)

In patients not being treated, 0.9% of patients had FT4 results outside the reference range (with 0.1% > 25 and 0.8% < 9). In 87% of these cases, their TSH was within reference range (0.4–3.9 miU/L).

In treated patients who only had single test in that year, that is, had no retests or dose adjustments:

- In total, 5.4% of patients had FT4 results outside the reference range (3.3% FT4 > 25pmol/L and 2.1% < 9pmol;/L). In 19% of these cases, their TSH values were within the TSH reference range (0.4–3.9 miU/L), that is, not concordant.
- In patients on lowest dose < 40 mcg/day Levothyroxine, 5.3% of patients had FT4 outside the reference range (0.9% > 25pmol/L and 4.4% < 9pmol/L) and in 28% of these cases, their TSH was within the reference range, that is, not concordant.
- In patients on the highest dose > 130 mcg/day Levothyroxine, 7.1% had FT4 outside the reference range (6.2% > 25pmol/L and 1.9% < 9pmol/L) 14% of these also had TSH outside the reference range, that is, not concordant.
- The proportion of patients with both normal range TSH and normal range FT4 was less in treated hypothyroid versus euthyroid individuals with the difference increasing with increasing LT4 dose.

## 4 Discussion

We have described here that the distribution of FT4/TSH is different in people on and off Levothyroxine treatment and that the degree of difference increases in treated individuals with Levothyroxine daily dose. The distribution of TSH is ‘unphysiological’ even at low Levothyroxine dose. Most Levothyroxine‐treated patients have an off‐target TSH level; this raises the question of how appropriate the Levothyroxine dose in many individuals is. For those on Levothyroxine, only 43.8% were within the TSH reference range and the degree of difference increased in treated individuals with Levothyroxine daily dose.

The data that we have been able to analyse do not take into account the circulating and tissue FT3 levels. FT3 levels are lower in the Levothyroxine‐treated patients. It is thought that a high FT4 (20 pM) may be needed to equate TSH levels in treated versus untreated individuals, possibly because at these FT4 levels, the FT3 levels are normal because of D1 activation [30]. With increasing LT4 dose, the ratio of FT4 to TSH rises. Furthermore, median TSH levels fell with increasing age in treated individuals.

The finding of lower median TSH and higher FT4 with increasing age in treated individuals may relate to a progressive increase in Levothyroxine dose in the years post‐diagnosis with hypothyroidism, which is not in accordance with recommended guidance. This runs counter to the principles of management of older people with treated hypothyroidism.

We also suggest that, given the unphysiological relation between TSH and FT4 in treated hypothyroid individuals, both FT4 and TSH should be measured for those being treated with Levothyroxine in order to enable effective clinical decision making. Notably, of the treated hypothyroid population, 2.5% of people had both a high TSH and high FT4, and 25.5% of individuals recorded a low TSH. This may be because of lower FT3 levels in these individuals [30]. In total, 10.0% of these were showing high FT4 values suggesting overreplacement.

The analyses in Figures 5 and 6, which look at the relation between FT4 and TSH in a reciprocal way by statistically dividing up the respective distributions and then looking at the effect of Levothyroxine dose on this relationship, provide further evidence for the very different relationship between FT4 and TSH in treated hypothyroid individuals versus untreated individuals with some modulation of the relationship by increasing Levothyroxine dose.

In a previous paper, Gullo et al. [14] reported that T4 levels were significantly higher and FT3 levels were significantly lower in 1811 Levothyroxine‐treated athyreotic patients (all had undergone total thyroidectomy) than in 3875 matched euthyroid controls. It was concluded that there is a highly heterogeneous T3 production capacity from orally administered Levothyroxine. In total, 20% of the people studied, despite normal TSH levels, did not maintain FT3 or FT4 values in the reference range. We have shown here similar findings at a larger population level, looking at 10.3 million TFTs in 1.1 million people over 14 years.

Tables 4 and 5 indicate the marked differences in the proportion in different categories of FT4/TSH—between hypothyroid‐treated and euthyroid individuals with implications for longer‐term thyroid‐related complications. We suggest that those patients who had discrepancies between TSH and FT4 classification should be carefully evaluated, as their condition could be more complex.

Timely Levothyroxine dose titration requires checking of thyroid hormone levels at recommended intervals. It is of note that previously, in a UK study, it was reported that the majority of TFTs that were checked were requested outside recommended intervals and that within‐general practice variability in test interval was high [31]. Specifically median re‐test interval was much lower than recommended (52 weeks) for those with normal TFTs at 31.3 weeks.

It is possible that our findings at a population level in relation to TSH and FT4 profile for Levothyroxine‐treated individuals reflect the inadequacy of peripheral deiodination to FT3 to compensate for the absence of Liothyronine endogenous production in Levothyroxine‐treated hypothyroid individuals. While most patients on Levothyroxine monotherapy will reach normal TSH serum levels, a normal FT3/FT4 ratio and also full improvement of signs and symptoms, a subset of patients is unable to convert the ingested Levothyroxine into an adequate amount of T3. This may happen for a variety of reasons, including congenital or acquired deficiency of deiodinase function [32, 33, 34] and also abnormal thyroid hormone metabolism independent from deiodination [35]. This can occur to different degrees in peripheral tissues and centrally in the hypothalamus, with the consequence that there may be ‘appropriate’ downregulation of TSH production at the anterior pituitary through downregulation of thyrotropin‐releasing hormone (TRH) while suboptimal peripheral deiodination occurs in the periphery [34].

The long‐term effects of chronic tissue exposure to an unphysiological T3/T4 ratio are unknown but the corollary would be that a more physiological treatment than Levothyroxine monotherapy may be required in some hypothyroid patients.

The finding that a proportion of individuals have both a high TSH and high FT4 may in some cases relate to ‘loading’ of Levothyroxine prior to the blood test for TFTs if people have not been fully concordant with Levothyroxine in the weeks before, leading to a high TSH (secondary to under replacement in the previous weeks) along with a high FT4 secondary to dose ‘loading’ prior to the TFT check. Furthermore, the time of Levothyroxine administration versus the time of the blood test can vary greatly [36].

There is also the potential for some individuals to be treated unnecessarily with Levothyroxine. We previously described the observation that a proportion of patients in the study that were on lithium treatment do return to euthyroidism if simply monitored [37]. There is, therefore, a case for not jumping to Levothyroxine treatment straight way but rather waiting to see the trend in TFTs over time, unless the patient is highly symptomatic.

We suggest that the implication of our findings is that clinicians must be mindful as they diagnose and treat hypothyroidism that the administration of Levothyroxine, while in most but not all individuals is clinically beneficial does not return the individual to the same balance of TSH and FT4 as seen in euthyroid individuals.

### 4.1 Limitations

We were not able to access sufficient FT3 data for the FT3 level to be factored into the analysis. Furthermore, the data are based exclusively on TFT results held in primary care and are from a heterogeneous group of hypothyroid patients, not just athyreotic individuals, some of whom may have had residual endogenous thyroid hormone secretion. We were able to analyse TFT results from 1.1 million individuals. We accept that this is from a subset of the England population. However, the population of Greater Manchester is representative of the population mix of England as a whole [38].

We did not have information about the timing of LT4 administration in relation to the blood test performed. Furthermore, we were not able to take account of specific patient‐level education at diagnosis and subsequently or concordance of the treated patients.

## 5 Conclusion

We have described here that the distribution of FT4/TSH is different in people on and off Levothyroxine treatment. For those on Levothyroxine, only 43.8% were within the TSH reference range and that the degree of difference increases in treated individuals with Levothyroxine daily dose. The distribution of TSH could be described as ‘unphysiological’ even at low Levothyroxine dose, highlighting the need for testing both TSH and fT4 to monitor efficacy of levothyroxine therapy. Furthermore, lower median TSH noted in older individuals suggests possible over‐replacement and a need to further examine the initial diagnosis leading to levothyroxine prescription. We hope that these observations will be helpful to clinicians.

## Author Contributions

Michael P. Stedman and Adrian H. Heald wrote the manuscript with data analysis by Michael P. Stedman. Lakdasa D. Premawardhana, Onyebuchi E. Okosieme, Peter N. Taylor, Antonio Bianco, Suhani Bahl, Anthony A. Fryer and Colin M. Dayan contributed to and have approved the final version of the manuscript. Anthony A. Fryer, Colin M. Dayan, Lakdasa D. Premawardhana, Antonio Bianco and Onyebuchi E. Okosieme provided senior review and editorial input.

## Funding

The authors received no specific funding for this work.

## Conflicts of Interest

The authors declare no conflicts of interest.

## Data Availability

Some or all data sets generated during and/or analysed during the current study are not publicly available but are available from the corresponding author on reasonable request.

## References

[1] A. Garmendia Madariaga , S. Santos Palacios , F. Guillén‐Grima , and J. C. Galofré , “The Incidence and Prevalence of Thyroid Dysfunction in Europe: A Meta‐Analysis,” Journal of Clinical Endocrinology and Metabolism 99, no. 3 (2014): 923–931. 24423323 10.1210/jc.2013-2409

[2] “National Institute of Diabetes Digestive and Kidney Disease; Hypothyroidism (Underactive Thyroid),” accessed December 24, 2024, https://www.niddk.nih.gov/health-information/endocrine-diseases/hypothyroidism#common.

[3] O. Leng and S. Razvi , “Hypothyroidism in the Older Population,” Thyroid Research 12, no. 1 (2019): 2. 30774717 10.1186/s13044-019-0063-3 PMC6367787

[4] L. Chaker , A. C. Bianco , J. Jonklaas , and R. P. Peeters , “Hypothyroidism,” Lancet 390, no. 10101 (2020): 1550–1562. 10.1016/S0140-6736(17)30703-1 PMC6619426 28336049

[5] D. S. Cooper and B. Biondi , “Subclinical Thyroid Disease,” Lancet 379, no. 9821 (2012): 1142–1154. 22273398 10.1016/S0140-6736(11)60276-6

[6] P. N. Taylor , M. M. Medici , A. Hubalewska‐Dydejczyk , and K. Boelaert , “Hypothyroidism,” Lancet 404, no. 10460 (October 2024): 1347–1364. 39368843 10.1016/S0140-6736(24)01614-3

[7] “National Institute for Health and Care Excellence; Thyroid Disease Assessment and Management,” accessed December 24, 2024, https://www.nice.org.uk/guidance/ng145/chapter/Recommendations#managing-primary-hypothyroidism. 31829541

[8] L. E. Braverman , S. H. Ingbar , and K. Sterling , “Conversion of Thyroxine (T4) to Triiodothyronine (T3) in Athyreotic Human Subjects,” Journal of Clinical Investigation 49, no. 5 (1970): 855–864. 4986007 10.1172/JCI106304 PMC535757

[9] A. Heald , M. Stedman , B. Okosieme , L. Premawardhana , P. Taylor , and C. Dayan , “Liothyronine Prescribing in England: Costs Versus Need,” Lancet 402, no. 10417 (2023): 2074–2075. 37979592 10.1016/S0140-6736(23)01792-0

[10] A. Heald , L. D. Premawardhana , P. N. Taylor , et al., “Liothyronine (LT3) Prescribing in England: Are Cost Constraints Inhibiting Guideline Implementation?,” Clinical Endocrinology 101, no. 1 (July 2024): 62–68. 38752469 10.1111/cen.15061

[11] M. Stedman , P. Taylor , L. Premawardhana , O. Okosieme , C. Dayan , and A. H. Heald , “Trends in Costs and Prescribing for Liothyronine and Levothyroxine in England and Wales 2011–2020,” Clinical Endocrinology 94, no. 6 (2021): 980–989. 33411974 10.1111/cen.14414

[12] W. M. Wiersinga , L. Duntas , V. Fadeyev , B. Nygaard , and M. P. J. Vanderpump , “2012 ETA Guidelines: The Use of L‐T4 + L‐T3 in the Treatment of Hypothyroidism,” European Thyroid Journal 1, no. 2 (2012): 55–71. 24782999 10.1159/000339444 PMC3821467

[13] P. Saravanan , W.‐F. Chau , N. Roberts , K. Vedhara , R. Greenwood , and C. M. Dayan , “Psychological Well‐Being in Patients on ‘Adequate’ Doses of L‐Thyroxine: Results of a Large, Controlled Community‐Based Questionnaire Study,” Clinical Endocrinology 57, no. 5 (2002): 577–585. 12390330 10.1046/j.1365-2265.2002.01654.x

[14] D. Gullo , A. Latina , F. Frasca , R. Le Moli , G. Pellegriti , and R. Vigneri , “Levothyroxine Monotherapy Cannot Guarantee Euthyroidism in all Athyreotic Patients,” PLoS One 6, no. 8 (2011): e22552. 21829633 10.1371/journal.pone.0022552 PMC3148220

[15] M. D. Ettleson , W. H. Prieto , P. S. T. Russo , et al., “Serum Thyrotropin and Triiodothyronine Levels in Levothyroxine‐Treated Patients,” Journal of Clinical Endocrinology & Metabolism 108, no. 6 (2022): e258–e266. 10.1210/clinem/dgac725 PMC10413428 36515655

[16] V. Panicker , P. Saravanan , B. Vaidya , et al., “Common Variation in the *DIO2* Gene Predicts Baseline Psychological Well‐Being and Response to Combination Thyroxine Plus Triiodothyronine Therapy in Hypothyroid Patients,” Journal of Clinical Endocrinology and Metabolism 94, no. 5 (2009): 1623–1629. 19190113 10.1210/jc.2008-1301

[17] G. J. Canaris , N. R. Manowitz , G. Mayor , and E. C. Ridgway , “The Colorado Thyroid Disease Prevalence Study,” Archives of Internal Medicine 160, no. 4 (2000): 526. 10695693 10.1001/archinte.160.4.526

[18] E. Agathoklis , M. Henry , D. Spinos , et al., “Adequacy of Thyroid Hormone Replacement for People With Hypothyroidism in Real‐World Settings: A Systematic Review and Meta‐Analysis of Observational Studies,” Clinical Endocrinology 100, no. 5 (2023): 488. 38037493 10.1111/cen.14998

[19] A. C. Bianco , Y. Bao , O. Antunez Flores , et al., “Levothyroxine Treatment Adequacy and Formulation Changes in Patients With Hypothyroidism: A Retrospective Study of Real‐World Data From the United States,” Thyroid 33, no. 8 (August 2023): 940–949. 37335236 10.1089/thy.2022.0382

[20] O. E. Okosieme , G. Belludi , K. Spittle , R. Kadiyala , and J. Richards , “Adequacy of Thyroid Hormone Replacement in a General Population,” QJM: An International Journal of Medicine 104, no. 5 (2010): 395–401. 21109503 10.1093/qjmed/hcq222

[21] M. Lillevang‐Johansen , B. Abrahamsen , H. L. Jørgensen , T. H. Brix , and L. Hegedüs , “Over‐ and Under‐Treatment of Hypothyroidism Is Associated With Excess Mortality: A Register‐Based Cohort Study,” Thyroid 28, no. 5 (2018): 566–574. 29631518 10.1089/thy.2017.0517

[22] R. Thayakaran , N. J. Adderley , C. Sainsbury , et al., “Thyroid Replacement Therapy, Thyroid Stimulating Hormone Concentrations, and Long Term Health Outcomes in Patients With Hypothyroidism: Longitudinal Study,” BMJ 366 (2019): l4892. 31481394 10.1136/bmj.l4892 PMC6719286

[23] M. T. Sheehan , “Biochemical Testing of the Thyroid: TSH Is the Best and, Oftentimes, Only Test Needed ‐ A Review for Primary Care,” Clinical Medicine & Research 14, no. 2 (June 2016): 83–92. 27231117 10.3121/cmr.2016.1309 PMC5321289

[24] A. H. Heald , L. D. Premawardhana , P. N. Taylor , et al., “How Does Thyroid Hormone Profile Differ on and Off Replacement Treatment?,” Clinical Endocrinology 102, no. 4 (April 2025): 490–495. 39702980 10.1111/cen.15185 PMC11874186

[25] “NHS Greater Manchester; Greater Manchester Care Record,” accessed August 21, 2024, https://gmwearebettertogether.com/gm-care-record/.

[26] “Health Innovation Manchester; Using the GMCR for secondary research,” accessed August 21, 2024, https://healthinnovationmanchester.com/thegmcarerecord/the-gm-care-record-for-secondary-uses-research.

[27] “NHS Digital SNOMED CT,” accessed April 30, 2025, https://digital.nhs.uk/services/terminology-and-classifications/snomed-ct.

[28] “NHS Digital; Read Codes,” accessed April 30, 2025, https://digital.nhs.uk/services/terminology-and-classifications/read-codes.

[29] “NHS Greater Manchester; Research and Planning,” accessed February 24, 2025, https://gmwearebettertogether.com/research-and-planning/.

[30] D. Salvatore , T. Porcelli , M. D. Ettleson , and A. C. Bianco , “The Relevance of T_3_ in the Management of Hypothyroidism,” Lancet Diabetes & Endocrinology 10, no. 5 (May 2022): 366–372. 35240052 10.1016/S2213-8587(22)00004-3 PMC9987447

[31] J. Scargill , M. Livingston , D. Holland , C. Duff , A. Fryer , and A. Heald , “Monitoring Thyroid Function in Patients on Levothyroxine. Assessment of Conformity to National Guidance and Variability in Practice,” Experimental and Clinical Endocrinology & Diabetes 125 (2017): 625–633. 28407667 10.1055/s-0043-103018

[32] J. Köhrle , F. Jakob , B. Contempré , and J. E. Dumont , “Selenium, the Thyroid, and the Endocrine System,” Endocrine Reviews 26 (2005): 944–984. 16174820 10.1210/er.2001-0034

[33] F. J. De Jong , R. P. Peeters , T. Den Heijer , et al., “The Association of Polymorphisms in the Type 1 and 2 Deiodinase Genes With Circulating Thyroid Hormone Parameters and Atrophy of the Medial Temporal Lobe,” Journal of Clinical Endocrinology & Metabolism 92 (2007): 636–640. 17105838 10.1210/jc.2006-1331

[34] V. Panicker , C. Cluett , B. Shields , et al., “A Common Variation in Deiodinase 1 Gene DIO1 Is Associated With the Relative Levels of Free Thyroxine and Triiodothyronine,” Journal of Clinical Endocrinology & Metabolism 93 (2008): 3075–3081. 18492748 10.1210/jc.2008-0397 PMC2515080

[35] T. J. Visser , J. C. J. van Buuren , M. Rutgers , S. J. Eelkman Rooda , and W. W. de Herder , “The Role of Sulfation in Thyroid Hormone Metabolism,” Trends in Endocrinology & Metabolism 1 (1990): 211–218. 18411120 10.1016/1043-2760(90)90055-8

[36] H. I. Jansen , A. Boelen , A. C. Heijboer , E. Bruinstroop , and E. Fliers , “Hypothyroidism: The Difficulty in Attributing Symptoms to Their Underlying Cause.” Frontiers in Endocrinology 14 (February 2023), 1130661, 10.3389/fendo.2023.113066. 36814580 PMC9939761

[37] H. L. Duce , C. J. Duff , S. Zaidi , C. Parfitt , A. H. Heald , and A. A. Fryer , “Evaluation of Thyroid Function Monitoring in People Treated With Lithium: Advice Based on Real‐World Data,” Bipolar Disorders 25, no. 5 (August 2023): 402–409. 36645255 10.1111/bdi.13298

[38] “Office of National Statistics; 2021 Census,” accessed December 20, 2024, https://www.ons.gov.uk/census.

